# Maternal chronic wasting disease infection restricts fetal head size in white-tailed deer (*Odocoileus virginianus*)

**DOI:** 10.1080/19336896.2026.2635296

**Published:** 2026-03-02

**Authors:** Jameson Mori, Sara Villazan Perez-Girones, Tooba Latif, Nelda Rivera, Dan Skinner, Peter Schlichting, Jan Novakofski, Nohra Mateus-Pinilla

**Affiliations:** a Illinois Natural History Survey, University of Illinois Urbana-Champaign, Champaign, USA; b Division of Wildlife Resources, Illinois Department of Natural Resources, Springfield, USA; c Department of Animal Sciences, University of Illinois Urbana-Champaign, Champaign, USA; d Department of Pathobiology, University of Illinois Urbana-Champaign, Urbana, USA; e Department of Natural Resources & Environmental Sciences, University of Illinois Urbana-Champaign, Urbana, USA

**Keywords:** Cervid, Cervidae, CWD, foetal development, prion, transmissible spongiform encephalopathy, TSE

## Abstract

Chronic wasting disease (CWD) is a fatal neurodegenerative prion disease of cervids that can be transmitted through direct physical contact, indirect contact with a contaminated environment, or vertical transmission. CWD is characterized by a long incubation period followed by symptoms like loss of appetite resulting from the destruction of brain tissue. While the consequences for infected animals are clear, a previous study from our laboratory showing lower body weights in the foetuses of CWD-positive female deer suggests those consequences may be intergenerational. In this study, we addressed the impact of maternal CWD infection on foetal head development (as a proxy for brain growth) in wild white-tailed deer using data from CWD management efforts in northern Illinois, U.S.A. Multivariate, multilevel, Bayesian Gamma regression found that maternal CWD infection reduced foetal head nose-occipital length and crown-jaw circumference by 6.76% and frontal-occipital length by 11.31%. These findings suggest impeded brain development in the offspring of CWD-infected female deer, which could reduce fawns’ survival and success after birth and lead to a decline in population fitness over time. This study is the first to demonstrate the detrimental effects of a prion disease on foetal brain development in any animal species regardless of whether vertical transmission has occurred.

## Introduction

Chronic wasting disease (CWD) is a prion disease affecting members of the family *Cervidae* caused by the misfolding of normal cellular prion proteins into non-functional aggregates in a neurodegenerative chain reaction that invariably results in death [1]. The incubation period is typically 1–2 years, with the symptomatic stage lasting a few months [1]. The characteristic signs of CWD infection include emaciation, behavioural changes, and loss of coordination [1], which are all caused by the progressive formation of non-functional protein aggregates in the brain that lead to a spongiform appearance and severe cognitive decline [2]. Symptomatic deer are rarely observed in the wild, however, because of the effectiveness of management efforts to remove CWD-positive animals from the landscape [3] and the increased risk of undocumented mortality from other causes [4].

CWD prions primarily replicate in nervous and lymphatic tissues [2] and have been detected in blood [5], saliva [6], urine [6], faeces [7], and reproductive tissues [8]. This broad tissue tropism facilitates the easy transmission of this pathogen through direct contact between animals [1] or exposure to contaminated soil [9], plants [10], or water [11]. Vertical transmission from mother to offspring *in utero* has also been documented [12], which raises questions about whether and how maternal CWD infection affects foetal development. A previous study by Mori et al [13] found that foetuses of pre-clinical CWD-positive female white-tailed deer (*Odocoileus virginianus*) weighed 1% less in the second trimester of gestation than those of CWD-negative females. Though the difference is small, fawns with lower body mass are at higher risk of mortality [14] and are less reproductively successful when they reach maturity [15]. Maternal age and body size are strong determinants of health and reproductive capacity, and by extension, foetal health and size [16], and so offspring of small females are likely to be small themselves. The cumulative effects of this cycle of transmission and foetal growth restriction at the population level could cause each generation to be less physically fit than the prior one and exacerbate the population declines predicted should CWD continue to increase in prevalence and spread geographically [17].

Skull size – and by extension, brain size – is one aspect of foetal growth that CWD may affect. Brain growth starts after the formation of the neural tube, with the skull beginning as a surrounding layer of flexible cartilage [18]. As the foetal brain increases in size, the skull expands along with it, with new cartilage created at zones commonly called ‘sutures’ and cartilage farther from these sutures converting to bone in response to a combination of biomechanical stress and cellular signalling [18]. Brain shape and size dictate the shape and size of the head, resulting in a direct relationship between their dimensions [18]. This means that smaller heads hold smaller brains and the cause of this decrease in size – relative to what is normal for the population – is reducing the amount of brain tissue in the foetus. Studies in humans have noted cognitive impairment associated with smaller heads and lower overall body weight, possibly due to a decrease in cortical volume, issues with myelination, and detrimental effects on neutrophils and the hippocampus [19]. Growth restriction in the brain thus raises concerns about survival, behaviour, and reproductive success of the affected animal.

Our study sought to determine whether maternal CWD infection alters foetal head size – as a reflection of brain size [20] – in wild white-tailed deer in northern Illinois, U.S.A. We conducted a multivariate, multilevel, Bayesian Gamma regression using data from 49 CWD-positive female deer (88 foetuses) and 1368 CWD-negative deer (2546 foetuses) collected in northern Illinois, U.S.A. Three head dimensions were examined: (1) nose-occipital length, (2) frontal-occipital length, and (3) crown-jaw circumference (Figure 3). It was hypothesized that the foetuses of CWD-positive female deer would have smaller head dimensions than the foetuses of CWD-negative deer.

## Results

### Principal component analysis(PCA)

**Figure 1.**
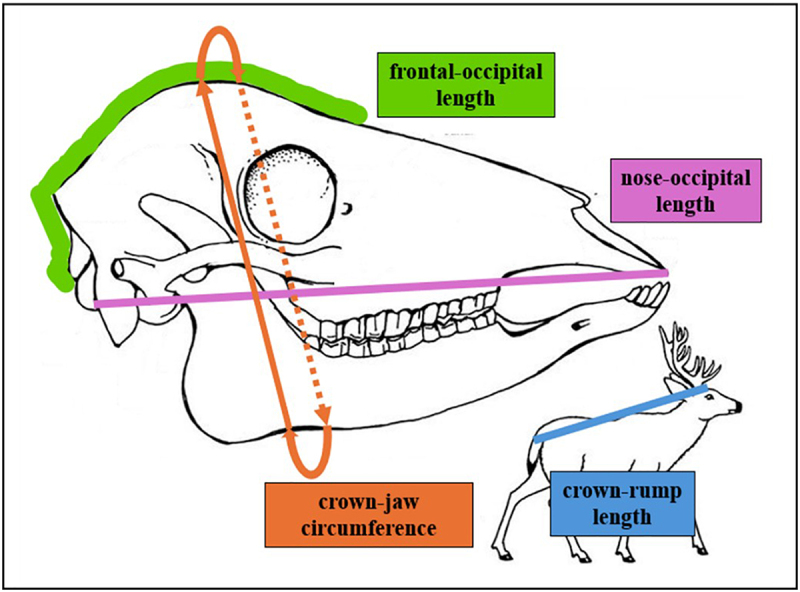
Scree plot of variance explained (y-axis) by each principal component (x-axis). The threshold for a significant amount of variance explained (>1) is indicated with a horizontal red line and significant principal components are marked with blue dots.

Due to the interrelatedness of the model covariates and interaction terms, we conducted principal component analysis (PCA) to distill information in these covariates into a set of uncorrelated principal components. All terms were included in this PCA except maternal CWD infection, as this was the variable of interest. A scree plot was used to identify significant principal components [21] (variance > 1) to retain as covariates for the regression model (Figure 1).

Principal components 1 through 3 had variances > 1. Table 1 shows the principal component loadings for each covariate and interaction term.

**Table 1.** Principal component (PC) loadings for each variable and interaction term.

Variable	PC1	PC2	PC3
Foetal sex	−0.01	−0.02	0.00
Foetal crown-rump length	**−0.39**	0.02	0.02
Foetal weight	**−0.43**	0.01	−0.01
Foetal weight [2]	**−0.41**	0.00	−0.03
Maternal age	−0.01	**0.52**	−0.04
Litter size	−0.02	0.15	**−0.47**
Day of culling	**−0.38**	−0.03	0.05
Maternal weight	0.04	**0.35**	**−0.35**
TRS land cover utility score (LCU)	0.00	0.14	**0.73**
Maternal age*Maternal weight	0.00	**0.54**	−0.11
Maternal age*LCU	0.00	**0.51**	**0.33**
Foetal weight*Foetal crown-rump length	**−0.43**	0.01	−0.01
Foetal weight [2]*Foetal crown-rump length	**−0.41**	0.00	−0.03
**Interpretation**	fetal development	maternal reproductive maturity	fetal environment

Loadings with a magnitude |>0.3| were considered meaningful (in bold). Negative loadings indicate an inverse relationship between the covariate and the principal component so that an increase in the variable corresponds to a decrease in the principal component value. The interpretation of the meaning of each principal component is also provided.

There are general trends in Table 1 that shed light on the interpretations of principal components 1–3. For principal component 1 (PC1), foetal crown-rump length, day of culling, both foetal weight terms, and related interaction terms had loading magnitudes >0.3, and so PC1 was interpreted as representing foetal development (Table 1). These PC1 loadings were all negative and therefore represented the ‘smallness’ of a foetus so that the larger the value of PC1 the smaller the foetus was. Maternal characteristics (age and weight) loaded most heavily on PC2, representing maternal reproductive maturity (Table 1). The loadings for PC2 were positive, meaning that larger PC2 values reflect more mature female deer. The loading magnitudes for litter size, maternal weight, and land cover utility score were >0.3, with litter size and maternal weight being negative and TRS LCU being positive (Table 1). A TRS (township, range, and section) is a ~2.6 km^2^ rectangle of land designated by the United States Public Land Survey System (US PLSS) [22] and is the smallest spatial unit at which CWD data is collected in Illinois [23]. PC3 May describe aspects of the environment, either *in utero* or in the external environment to which the female deer is exposed. However, the exact relationships among the variables in PC3 are unclear.

### Level selection

The intraclass correlation coefficient (ICC) was calculated for four potential data groupings (levels) – unique female deer identifier (Doe_ID), TRS, County, and fiscal year (FY) – to evaluate their ability to describe the variation in the data. An ICC value of 1 indicates that foetuses within the same group are highly related and a value of 0 indicates no relation [24]. The female deer identifier had ICC’s indicating strong relationships between the head measurements of foetuses in the same litter, with the ICC of nose-occipital length equal to 0.812, frontal-occipital length equal to 0.808, and crown-jaw circumference equal to 0.843. The other three levels – TRS, County, and FY – explained very little of the variance. The ICCs for TRS were 0.027, 0.016, and 0.023 for nose-occipital length, frontal-occipital length, and crown-jaw circumference, respectively, 0.005, 0.006, and 0.006 for County, and 0.027, 0.026, and 0.021 for fiscal year.

### Multivariate multilevel Bayesian Gamma regression

Multivariate multilevel Bayesian Gamma regression of foetal head dimensions showed that maternal CWD infection, PC1 (foetal development), and PC2 (maternal reproductive maturity) were statistically significant for all foetal head dimensions (Table 2). Statistical significance is defined as the regression covariate having a 95% credibility interval that does not contain 0 [25]. PC3 (environment) was also significant for frontal-occipital length (Table 2).

**Table 2.** Multivariate multilevel Bayesian Gamma regression of the impact of maternal infection with chronic wasting disease (CWD) and principal components reflecting foetal (PC1), maternal (PC2), and environmental factors (PC3) on foetal head dimensions.

Foetal Head Dimension	Variable	Relative Rank	Regression Coefficient	95% CI		Change (%)
				LB	UB	
Nose-occipital length	CWD	2	−0.07	−0.12	−0.02	−6.76
	PC1	1	−0.49	−0.51	−0.47	−38.74%
	PC2	3	0.05	0.03	0.06	5.13%
Frontal-occipital length	CWD	2	−0.12	−0.17	−0.07	−11.31%
	PC1	1	−0.44	−0.46	−0.42	−35.60%
	PC2	3	0.03	0.02	0.05	3.05%
	PC3	4	0.02	0.01	0.04	2.02%
Crown-jaw circumference	CWD	2	−0.07	−0.13	−0.02	−6.76
	PC1	1	−0.44	−0.46	−0.42	−35.60%
	PC2	3	0.06	0.04	0.08	6.18%

Coefficients are statistically significant if 0 does not fall between the lower (LB) and upper bounds (UB) of the 95% credibility interval (CI). The percent change in foetal head dimensions when a female deer is CWD-positive was calculated from the regression coefficient using the equation: [Exp(coefficient)-1]*100. Negative coefficients and percentages indicate that when the covariate increases, the foetal head dimension decreases.

## Discussion

The multivariate multilevel Bayesian Gamma regression found that maternal infection with chronic wasting disease significantly reduced all three foetal head dimensions examined in this study: (1) nose to occipital length, (2) frontal to occipital length, and (3) crown to jaw circumference. Given that the amount of brain tissue dictates the size of the skull during foetal development [18], it can be inferred that CWD is in some way impairing brain growth in white-tailed deer. It bears repeating that the inclusion of foetal body weight and length in the principal components used in this regression model means that this decrease in brain size accounts for the relationship between body and head size, and that the brains of CWD-positive females are smaller than would be expected given their body size.

When looking specifically at the model results, it can be observed that the statistically significant covariate with the most influence on foetal head dimensions was PC1, which had negative relationships with all three head measurements (Table 2). Given that PC1 represents foetal ‘smallness’, this suggests that foetuses with larger PC1 values (smaller body size) have smaller heads, accurately reflecting the general proportionality of foetal growth (Table 2). PC2 was consistently the third most important variable, showing a positive relationship with foetal head dimensions to indicate that more mature female deer have larger offspring (Table 2). PC3 was the least important of the significant variables (Table 2).

The second most important variable explaining all three foetal head dimensions was maternal CWD infection (CWD). The regression revealed that foetal nose-occipital length and crown-jaw circumference in foetuses of CWD-positive deer were both 6.76% smaller than those of foetuses from CWD-negative deer. Similarly, frontal-occipital length was 11.31% smaller in foetuses of CWD-infected females (Table 2). Not only does this mean that CWD is detrimental to foetal development, but it ranks maternal CWD infection as more influential than maternal reproductive maturity or environmental factors (Table 2). This means that CWD infection is a stronger determinant of foetal head size than maternal age or body weight, two factors well documented to impact foetal size [16]. The observation that all three head measurements were significant, negative, and close in value suggests a mechanism that restricts brain size as a whole rather than affecting only certain regions of the brain.

The identification of a negative impact of CWD on foetal brain development then raises the question of how CWD can have this impact. We propose four hypotheses to explain how maternal CWD infection, either working separately or in tandem. The first reason for decreased foetal head dimensions could be nutrient restriction caused by neuroinflammation [26] or a decreased ability to absorb vital nutrients due to alterations in the gut microbiome observed in CWD-affected animals [27]. Maternal body weight itself is controlled for in the model, but it is possible that the infection has begun to cause nutrition deficiencies even before physical wasting starts. The detrimental effects of nutritional deficiencies on foetal neurological development are well known in humans [28], but to our knowledge, nutrient restriction in CWD-infected cervids has not been evaluated beyond the observation of physical wasting. It is therefore unknown when the decrease in nutrient uptake begins, but the findings of this study suggest this impairment may begin before the onset of clinical signs. Neuroinflammation itself is the second hypothesis proposed to explain reduced foetal head size. The inflammation caused by neurodegeneration in the infected female [29] creates physiological stress for the developing foetus. Prion diseases induce inflammation through the accumulation of cytotoxic aggregates and increased production of pro-inflammatory microglial cells and astrocytes [29]. Studies in humans have observed impaired neurodevelopment in foetuses of mothers experiencing neuroinflammation [29] and it is possible that the same process occurs in cervids.

The third reason for decreased foetal head dimensions could be that vertical transmission of the pathogenic prion from the infected female to the foetus directly interferes with brain growth. While the roles of the normal prion protein in the body are not fully understood, we do know that it is a cell surface protein found most abundantly in nervous system tissues [30]. Expression of the normal prion protein has been observed soon after embryonic implantation and throughout foetal development, and various studies have identified possible roles in early stem cell differentiation, cell adhesion, and formation of neurons, astrocytes, and glial cells [30]. The normal prion protein may also help with maintaining the structure and function of synapses [30], modulating glutamate receptors [31], and regulating the growth of axons [30]. The ubiquity and early expression of this protein could make foetuses more susceptible to acquiring the prion infection through the maternal-foetal interface. It should be noted that the foetuses involved in this study were not themselves tested for CWD infection due to uncertainties regarding the accuracy of pathogenic prion detection at such low concentrations, so it is unknown whether vertical transmission occurred. The fourth possibility is that foetal exposure to the pathogenic prions is too low to cause infection, but sufficient to impede normal development by slowing formation of key cells and structures. Regardless of the mechanisms at play, ours is the first study to detect a negative impact of pre-clinical maternal prion disease on foetal brain development, whether or not vertical transmission has occurred.

These results, when combined with our previous finding of lower foetal body weights in foetuses from CWD-positive females [13], inspire questions of consequences. At the individual level, smaller heads mean smaller brains, and smaller brains could worsen symptoms already associated with CWD, such as reduced cognitive ability [1]. Studies in mice also suggest that smaller brains may lead to faster mortality since less tissue means fewer normal prion proteins to misfold [32]. Thus, at the individual level, these effects make for a shorter and harder life.

At the population level, CWD threatens to reduce herd fitness over time as the number of infected animals increases. Deer have a prodigious reproductive capacity for a large mammal, producing litters ranging in size from 1 to 5 foetuses annually [33] (median = 2), and studies have found no reduction in pregnancy rates associated with CWD infection [34]. This turns reproduction into a series of events that spread the disease between females and males during the breeding season, and then between females and offspring during gestation and early life. The population effects are thus exponential, especially when the role of indirect transmission via a contaminated environment is also considered, which only worsens as the cumulative presence of CWD-infected animals on the landscape increases [35,36]. In line with the predictions of statistical models [36], this increase in CWD burden could lead to population reductions to the point of collapse. The severity of these impacts emphasizes the importance of the efforts of the IDNR, deer hunters, and landowners for actively managing CWD in affected areas of Illinois to interrupt transmission cycles, slow geographic spread, and reduce prevalence.

The main limitations of our study are threefold, with the biggest being that foetal brain size was approximated with skull size instead of being measured directly because of the complete loss of tissue integrity upon removal. The brains of foetuses in the late third trimester or neonates immediately after birth should maintain their structure better, which would also address another limitation of this study, which is that the foetuses examined were in their second trimester of development. Though our findings can be used to hypothesize effects later in development, this dataset does not cover the entirety of gestation and therefore the relationships observed may change as foetuses continue to grow. Lastly, the population evaluated in this study was limited to northern Illinois, and so the ability to extrapolate these findings to different populations – especially different cervid species – remains unknown. Despite these limitations, this study establishes the need to investigate this subject further in both cervids and other species affected by prion diseases.

## Methods

All analyses were conducted in R (v. 4.4.3) [37]. The data sets used herein are available in the *Supplementary Materials*.

### Study area

**Figure 2.**
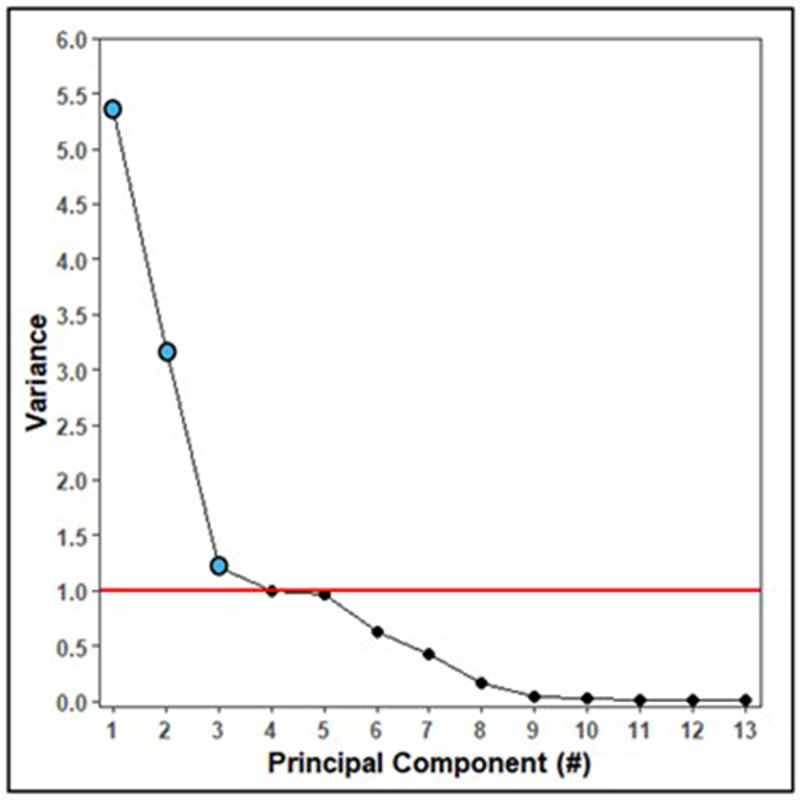
Map of study area. The counties (grey) and TRS’ (township, range, and section; blue) in northern Illinois, USA from which the pregnant female deer in the dataset for this study originated from. The total number of female deer from each county is listed in the rightmost panel, with each county in the map labelled with a number to link it to the list.

The dataset utilized in this study contained measurements from wild white-tailed deer culled by the Illinois Department of Natural Resources (IDNR) as part of their ongoing chronic wasting disease (CWD) management efforts [23]. This dataset included 88 foetuses from 49 CWD-positive deer and 2546 foetuses from 1386 CWD-negative deer. Deer were obtained from 239 TRS’ (township, range, and section) in 18 counties in northern Illinois, U.S.A. between fiscal years 2018 and 2024 (Figure 2). Culling occurs between January and March of each fiscal year [23], which means these foetuses were in the second trimester of development. A fiscal year (FY) is the period between July 1 of one calendar year and June 30th of the next. The IDNR reports CWD data by FY because it groups management actions (hunting and culling) and deer biology (breeding in Fall and fawning in Spring) together in the same time period.

### Foetal head dimensions

**Figure 3.**
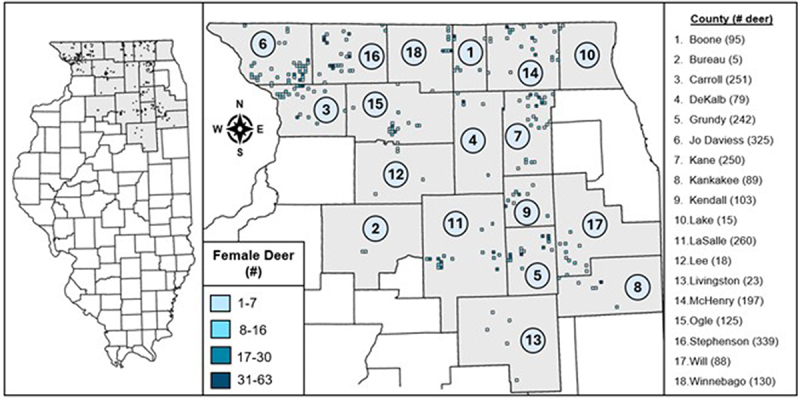
White-tailed deer(*Odocoileus virginianus*) skull and body measurements.

Three foetal head dimensions were of interest in this study: (1) the length from frontal to occipital bone (FO), (2) the length from the nose to the occipital bone (NO), and (3) the circumference from the top of the head (crown) to the underside of the jaw (CJ) (Figure 3).

**Figure 4.**
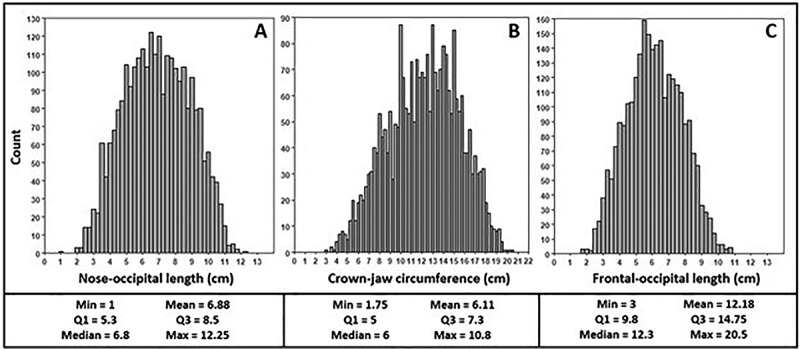
Histograms showing the distributions of foetal nose-occipital length (A), crown-jaw circumference (B), and frontal-occipital length (C). Summary statistics are listed in the top right portion of each panel.

The histograms in Figure 4 show the distributions of values for each foetal head measurement.

### Model covariates

**Figure 5.**
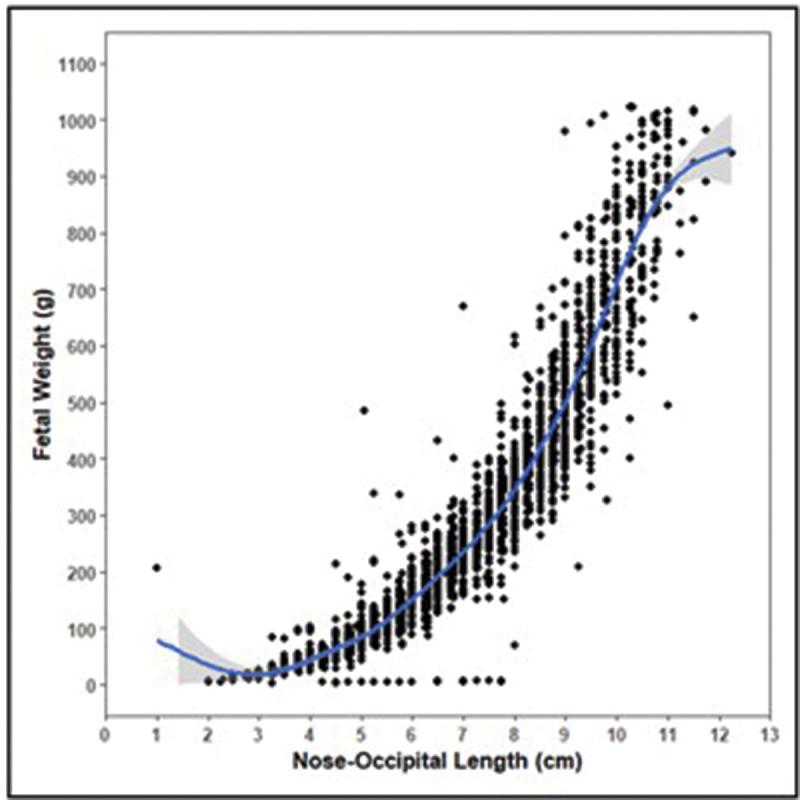
The weight of each foetus plotted in relation to the nose-occipital length with a smoothed, loess line (blue) to highlight the general trend.

Foetal sex was a binary variable representing whether the foetus was female (0) or male (1). The relationship between foetal body and head size was accounted for via the inclusion of foetal body weight and crown-rump length as covariates. A squared foetal weight term was included as well because of the nonlinear relationship between foetal weight and foetal head dimensions (Figure 5).

Maternal body weight was measured immediately after culling because it is known to have significant effects on deer health and reproduction, as well as foetal body weight, with smaller does tending to have smaller foetuses [16]. Maternal age was estimated using tooth wear [38] and was included because adult females tend to produce more and larger offspring [16]. The day of culling was used in the model as a proxy for duration of gestation, with day 1 being July 1^st^ and day 365 being June 30^th^. Litter size was included because twins tend to be smaller than singletons, with triplets being even smaller [31]. The land cover utility (LCU) score of the TRS was included from previous work by Mori et al [39] to account for the effects of habitat on deer health and reproductive characteristics [40].

In addition to the singular covariates, there were four interaction terms included in the model: (1) maternal age and weight, (2) maternal age and LCU, (3) foetal weight and crown-rump length, and (4) foetal weight [2] and crown-rump length. It was suspected that maternal weight would influence the effect of maternal age on foetal development, with younger but heavier deer producing foetuses closer in size to older deer. Deer land cover utility (LCU) score was believed to effect reproduction in younger females more than older females, with younger females producing more and larger foetuses when in TRS’ with abundant, high-quality habitat as represented with a high LCU score. This decision was supported by findings from Mori et al. (2025), which found that fawns in TRS’ with higher LCU scores were more likely to become pregnant, but this effect did not extend to yearlings or adults. An interaction between foetal weight and crown-rump length was considered because together they represent the growth of the foetuses’ body. Summary statistics for all model covariates are provided in Table 3.

**Table 3.** Covariates, units of measurement, and values present in the dataset used for modelling foetal head size in wild white-tailed deer(Odocoileus virginianus).

Variable	Units	Values		
		Min	Median	Max
Nose-occipital length (NO)	cm	1	6.8	12.25
Crown-jaw circumference (CJ)	cm	3	12.3	20.5
Frontal-occipital length (FO)	cm	1.75	6	10.8
Foetal sex	-	female (0) or male (1)		
Foetal crown-rump length	cm	3.25	17.75	32
Foetal weight	g	3	221.7	1022.5
Maternal age^a^	years	0	2	5
Maternal weight	kg	37	61.4	84.6
Litter size	fetuses	1	2	3
Day of culling	day	18	47	88
Maternal CWD infection status	–	not detected (0) or positive (1)		
TRS land cover utility (LCU) score	–	1141	8633	10,770

^a^Female fawns were considered to be age ‘0’.

### Principal component analysis(PCA)

The covariates and interaction terms were highly interrelated, which meant they could not be included in the same model without causing instability in the regression coefficients. To circumvent this issue, the information contained in these variables was distilled into a set of uncorrelated variables using principal component analysis (PCA). PCA was conducted using the base R function ‘prcomp’ with both ‘center’ and ‘scale’ equal to ‘TRUE’.

To determine the appropriate number of principal components to use in the analysis, a scree plot was generated that shows how much variance is explained by each principal component (Figure 1). Principal components were considered significant if their explained variance was >1 and their scores were retained as covariates.

### Data centring and scaling

Prior to use in the regression, the principal components were mean centred and scaled by dividing by twice their standard deviations, while maternal CWD status was mean centred [41]. This was done to allow direct comparison between covariates and reduce model convergence time [41].

### Level selection

The appropriate levels to include in the analysis were determined by calculating the intraclass correlation coefficient (ICC) for each potential grouping factor – unique female deer identifier (Doe_ID), County and TRS of culling, and fiscal year (FY). Three Bayesian Gamma regressions were run without covariates using the ‘glmer’ function in the ‘lme4’ package [42] (v. 1.1–37), each modelling one of the foetal head dimensions. Gamma regression with a ‘log’ link function was done because all three outcome variables were continuous and strictly positive.

The ICC for each potential level in the three models was then obtained using the ‘icc’ function in the ‘performance’ (v. 0.15.1) R package [43]. ICC values were calculated by group. Levels with large values (close to 1) were selected for inclusion in all subsequent regressions [24]. *Equation 1* shows the model structure for this level selection process, with nose-occipital length (NO), frontal-occipital length (FO), and crown-jaw circumference (CJ) as simultaneous outcomes.

(1) $$\mathrm{NO},\;\mathrm{FO},\;\mathrm{or}\;\;\mathrm{CJ}\;∼\;1\;+\;\left(1|\mathrm{Doe}\_\mathrm{ID}\right)\;+\;\left(1|\mathrm{County}/\mathrm{TRS}\right)\;+\;\left(1|\mathrm{FY}\right)$$

### Multilevel multivariate Bayesian Gamma regression

A multilevel multivariate Bayesian Gamma regression was conducted using the ‘brms’ package [44] (v. 2.23.0) in R with foetal nose-occipital length, frontal-occipital length, and crown-jaw circumference as the dependent variables. This approach was chosen to allow isolation of the effect of maternal CWD infection on foetal head dimensions by controlling for factors that also impact head size such as maternal and foetal body weight, foetal length, maternal age, and foetal sex. A multivariate analysis was selected – which models the three foetal head dimensions together – because these measurements are strongly correlated and modelling them in separate regressions would ignore these dependencies and yield inaccurate results.

Maternal CWD infection was the variable of interest in this model, and the principal components and interaction terms were the covariates. Gamma regression with a log link function was chosen because all foetal head dimensions are positive and continuous. The prior distribution for all regression covariates was a normal distribution with a mean of 0 and a standard deviation of 0.25, both of which were obtained from the centred and scaled covariate values. The default priors for the regression intercepts and random effects were used [44].

The model was set to run for a total of 35,000 iterations, with the first 20,000 iterations discarded as burn-in to get a net total of 15,000 iterations [45]. The model was run with 8 chains on 8 cores. The thinning parameter was set equal to 4 to reduce autocorrelation between consecutive samples and reduce computational demands [46]. To ensure a sufficient sample size [43] (>0.1), the minimum effective sample size was calculated using the ‘neff_ratio’ function in the ‘performance’ package (v. 0.15.1) [47]. The ‘rhat’ function from ‘performance’ was used to confirm model convergence with an Rhat statistic close to 1^47^. The model structure for the first round of regression is provided in Equation (2), with interactions indicated with asterisks.

(2) $$\mathrm{NO},\;\mathrm{FO},\;\mathrm{and}\;\;\mathrm{CJ}\;∼\;\mathrm{CWD}+\mathrm{PC}1+\mathrm{PC}2+\mathrm{PC}3+\;\mathrm{CWD}*\mathrm{PC}1+\mathrm{CWD}*\mathrm{PC}2+\mathrm{CWD}*\mathrm{PC}3+\left(1|\mathrm{Doe}\_\mathrm{ID}\right)$$

The regression coefficients of all covariates and interaction terms from the first round of regression modelling were examined for statistical significance, defined as the 95% credibility interval not containing 0 [48]. Statistically significant covariates and interaction terms were retained for a second round of regression modelling. All results were obtained from this second model. This resulted in three substructures to the regression, shown in *Equations 3-5*.

(3) $$\mathrm{NO}\;∼\;\mathrm{CWD}\;+\;\mathrm{PC}1\;+\;\mathrm{PC}2\;+\;\left(1|\mathrm{Doe}\_\mathrm{ID}\right)$$

(4) $$\mathrm{FO}\;∼\;\mathrm{CWD}\;+\;\mathrm{PC}1\;+\;\mathrm{PC}2\;+\;\mathrm{PC}3\;+\;\left(1|\mathrm{Doe}\_\mathrm{ID}\right)$$

(5) $$\mathrm{CJ}\;∼\;\mathrm{CWD}\;+\;\mathrm{PC}1\;+\;\mathrm{PC}2\;+\;\left(1|\mathrm{Doe}\_\mathrm{ID}\right)$$

## Supplementary Material

Supplemental Material

## Data Availability

The authors confirm that the data supporting the findings of this study are available in the article’s supplementary materials.
