# Supplementary material for: Maternal chronic wasting disease infection restricts fetal head size in white-tailed deer (Odocoileus virginianus)
Source: Prion. 2026 Mar 2;20(1):18–30. doi: 10.1080/19336896.2026.2635296 (PMC12959197; doi:10.1080/19336896.2026.2635296)
Supplement: Supplemental Material [file KPRN_A_2635296_SM2850.docx]

**Supplementary Materials**

*Supplementary Dataset 1*

Anonymized dataset containing all dependent and independent variables in their original units prior to principal component analysis. The location and date of female deer culling is also provided.

*Supplementary Dataset 2*

Anonymized dataset used in the multivariate, multilevel, Bayesian Gamma regression. Contains all dependent variables, maternal CWD infection status, principal components, and location/date information for female deer. Maternal CWD status and principal components are mean centered and scaled by dividing by twice their standard deviations.
